# *MARC1* p.A165T variant is associated with decreased markers of liver injury and enhanced antioxidant capacity in autoimmune hepatitis

**DOI:** 10.1038/s41598-021-03521-3

**Published:** 2021-12-23

**Authors:** Maciej K. Janik, Wiktor Smyk, Beata Kruk, Benedykt Szczepankiewicz, Barbara Górnicka, Magdalena Lebiedzińska-Arciszewska, Yaiza Potes, Inês C. M. Simões, Susanne N. Weber, Frank Lammert, Mariusz R. Więckowski, Piotr Milkiewicz, Marcin Krawczyk

**Affiliations:** 1 Liver and Internal Medicine Unit, Department of General, Transplant and Liver Surgery, Medical University of Warsaw, Warsaw, Poland; 2 European Reference Network on Hepatological Diseases (ERN RARE-LIVER), Hamburg, Germany; 3 Laboratory of Metabolic Liver Diseases, Department of General, Transplant and Liver Surgery, Centre for Preclinical Research, Medical University of Warsaw, Warsaw, Poland; 4 Department of Pathology, Medical University of Warsaw, Warsaw, Poland; 5 Laboratory of Mitochondrial Biology and Metabolism, Nencki Institute of Experimental Biology Polish Academy of Sciences, Warsaw, Poland; 6 Department of Medicine II, Saarland University Medical Center, Saarland University, Homburg, Germany; 7 Hannover Medical School, Hannover, Germany; 8 Translational Medicine Group, Pomeranian Medical University, Szczecin, Poland

**Keywords:** Genetics, Hepatology

## Abstract

The clinical picture of autoimmune hepatitis (AIH) varies markedly between patients, potentially due to genetic modifiers. The aim of this study was to evaluate genetic variants previously associated with fatty liver as potential modulators of the AIH phenotype. The study cohort comprised 313 non-transplanted adults with AIH. In all patients, the *MARC1* (rs2642438), *HSD17B13* (rs72613567), *PNPLA3* (rs738409), *TM6SF2* (rs58542926), and *MBOAT7* (rs641738) variants were genotyped using TaqMan assays. Mitochondrial damage markers in serum were analyzed in relation to the *MARC1* variant. Carriers of the protective *MARC1* allele had lower ALT and AST (both P < 0.05). In patients treated for AIH for ≥ 6 months, *MARC1* correlated with reduced AST, ALP, GGT (all P ≤ 0.01), and lower APRI (P = 0.02). Patients carrying the protective *MARC1* genotype had higher total antioxidant activity (P < 0.01) and catalase levels (P = 0.02) in serum. The *PNPLA3* risk variant was associated with higher MELD (P = 0.02) in treated patients, whereas *MBOAT7* increased the odds for liver cancer (OR = 3.71). None of the variants modulated the risk of death or transplantation. In conclusion, the *MARC1* polymorphism has protective effects in AIH. Genotyping of *MARC1*, *PNPLA3,* and *MBOAT7* polymorphisms might help to stratify patients with AIH.

## Introduction

Autoimmune hepatitis (AIH) is a progressive liver disease, which, if untreated, leads to liver cirrhosis^1^. Therapy for AIH is based on immunosuppression, which induces remission in the majority of patients; some patients may require lifelong maintenance therapy or treatment with alternative, second-line drugs^1^. The progression of AIH varies markedly between patients^2^, and up to 30% of patients have liver cirrhosis at the time of diagnosis.

In the last decade, several inherited determinants of liver injury were identified^3^. For example, the common *PNPLA3* p.I148M, *MBOAT7* p.G17E, and *TM6SF2* p.E167K variants have been shown to increase the risk of liver steatosis, fibrosis, and cirrhosis in patients with nonalcoholic fatty liver disease (NAFLD), alcoholic liver disease (ALD), and with viral liver diseases^3–6^. Recently, two variants, *HSD17B13* rs72613567:TA^7^ and *MARC1* p.A165T^8–10^, were shown to decrease the risk of developing NAFLD. The *MARC1* p.A165T variant was demonstrated to have protective effects in patients with alcoholic liver disease (ALD)^11^. Rare liver diseases, such as AIH, have not been thoroughly studied in terms of genetic modifiers of their progression. A genome-wide association study (GWAS) in 649 adults with AIH from the Netherlands identified human leukocyte antigen (HLA) loci, HLA-DRB1*0301 and HLA-DRB1*0401, as susceptibility genotypes^12^, which was in line with other genetic studies on AIH^13–15^. The strongest non-HLA susceptibility marker was found to be *SH2B3*^12^, which was also reported to increase the risk of developing other autoimmune diseases, such as primary sclerosing cholangitis (PSC)^16^, primary biliary cholangitis (PBC)^17^, and type 1 diabetes mellitus^18^. Analyses of PBC and PSC risk loci in patients with AIH without cholestatic variants revealed that part of the genetic susceptibility for AIH coincides with PBC and PSC^12^. Other non-HLA susceptibility polymorphisms, for example, tumor necrosis factor alfa (TNF-alpha)^19^ or vitamin D receptor (VDR)^20,21^, were also associated with AIH in the previous studies. A recently published single-center analysis of 239 adult patients with AIH demonstrated that carriers of the minor *PNPLA3* p.I148M allele are characterized by advanced liver fibrosis according to the AST to Platelet Ratio Index (APRI) and Fibrosis-4 Index (FIB-4)^22^. Furthermore, it was shown that the *PNPLA3* p.148MM genotype might increase the odds of liver transplantation or death in patients with AIH^22^; however, these results have not yet been validated in other cohorts of patients with AIH.

In our current study, we investigate the role of inherited predisposition in the progression of liver injury in patients with AIH. To this end, we genotyped *PNPLA3*, *MBOAT7*, *TM6SF2*, *HSD17B13*, and *MARC1* variants in a large cohort of adult patients with AIH and performed the genotype–phenotype analyses.

## Patients and methods

Between October 2015 and June 2019, we prospectively enrolled 313 consecutive non-transplanted adults with AIH at the Medical University of Warsaw, Poland. Pure AIH, as well as its cholestatic variants, namely AIH-PSC and AIH-PBC, were diagnosed according to the current guidlines^1^ (i.e., they were based on liver histology, presence of typical antibodies, elevated serum concentration of immunoglobulin G, and, in cholestatic variants, imaging studies). The diagnosis of AIH-PSC overlap was based on imaging or histological features of PSC combined with features (biochemical, serological, and histological) of AIH. AIH-PBC diagnosis was established according to the “Paris criteria”^23^. Any acute or chronic liver diseases other than AIH, or its overlap with PSC and PBC, served as exclusion criteria.

The study protocol (KB/128/2015) was approved by the Ethics Committee of the Medical University of Warsaw, according to the ethical guidelines of the 1975 Declaration of Helsinki (latest revision, 2013), and written informed consent was obtained from all participants.

### Clinical data

All patients underwent careful clinical examination. Blood samples were drawn from fasted subjects. Non-invasive measurements for liver fibrosis and blood tests were performed in all patients on the same day, i.e., upon enrolment in the study. Liver fibrosis was non-invasively quantified using real-time 2D-SWE by Aixplorer (SuperSonic Imagine, Aix-en-Provence, France), as described previously^24^, and is presented as LSM. The following serum fibrosis indices were calculated as follows: FIB-4 was calculated according to Sterling et al.^25^, and the APRI was calculated in line with Wai et al.^26^. Liver biopsies were available in 48 (80%) of therapy-naïve patients; LB was not performed in 12 (20%) of therapy-naïve patients due to ascites, coagulopathy, or lack of patient consent. Biopsies were analyzed by a pathologist experienced in liver diseases using the Batts and Ludwig^27^ system for the assessment of liver fibrosis and inflammation. The available clinical data from the follow-up were used to examine changes in blood tests in relation to the studied polymorphisms.

Given the differences between pure AIH and its cholestatic variants, we performed genotype–phenotype association tests in the entire cohort of patients and separately in subsets of patients with pure AIH and with AIH-PSC or AIH-PBC. The subgroup of patients with pure AIH was divided in relation to the immunosuppressive therapy (i.e., therapy ≥ 6 months or therapy-naïve).

### Data at diagnosis, clinical outcome, and hepatocellular carcinoma

In addition to the data collected at baseline, we analyzed the association between the studied polymorphisms and (1) age and presence of liver cirrhosis at diagnosis, as well as the type of AIH; (2) clinical outcome defined as a combined endpoint: liver transplantation (LT) or liver-related death; and (3) hepatocellular carcinoma (HCC). Due to the relatively small number of patients who reached the clinical endpoint in the study group (n = 66, 21%), the additional cohort of 30 patients after LT for AIH (57% female, median age at diagnosis 33 (range 8–62) years, median age at LT 35 (range 12–67) years) was included in this analysis. The diagnosis of HCC was established by imaging studies (CT or MRI) and confirmed by histology, if available.

### Genotyping

Genotyping of the *MARC1* (rs2642438), *PNPLA3* (rs738409), *TM6SF2* (rs58542926), *MBOAT7* (rs641738), and *HSD17B13* (rs72613567) gene variants was performed at Saarland University Medical Center in Homburg, Germany, by technicians blinded to the phenotype of patients. A detailed description of genotyping is provided in Supplementary Text S1 and Table S1.

### Hepatocyte mitochondrial damage in relation to the *MARC1* genotype

How *MARC1* may impact liver damage is unclear; however, this signal-anchored mitochondrial protein^28^ is associated with detoxification reactions^29^. Thus, we evaluated the presence of oxidative stress-induced mitochondrial damage markers of hepatocytes in serum. The level of mitochondrial uncoupling protein 2 (UCP2) (an inner mitochondrial membrane marker) and two antioxidant enzymes, thioredoxin reductase 2 (TrxRd2) and superoxide dismutase 2 (SOD2) (mitochondrial matrix markers), in the serum of 39 individuals carrying either the wild-type (n = 25) or the homozygous (n = 14) *MARC1* variant were evaluated. This cohort was randomly selected from the pure AIH group (without AIH overlap with PSC and PBC) treated for at least 6 months. Patients for this analysis were randomly selected from the AIH cohort using SPSS software (IBM Corp. Released 2020. IBM SPSS Statistics for Windows, Version 27.0. Armonk, NY: IBM Corp.).

### Western blot analysis

The presence of antioxidant enzymes: ubiquitous catalase (CAT) and mitochondrially located TrxRd2 and SOD2, or mitochondrial proteins like UCP2 in serum, which could be linked to damage of hepatocytes, was evaluated with the use of specific antibodies, as described in detail in Supplementary Text S2.

### Determination of oxidative damage markers in serum

Lipid peroxidation (LPO) in serum was evaluated by measuring malondialdehyde (MDA) (a major reactive aldehyde resulting from the peroxidation of biological membranes) using the Lipid Peroxidation Assay Kit from Abcam (ab118970, Cambridge, U.K.). Serum protein oxidative damage was estimated using OxyBlot Protein Oxidation Detection Kit (S7150, Sigma-Aldrich, MO, USA). A detailed description of the performed experiments is provided in Supplementary Text S2.

### Serum total antioxidant activity

The concentration of small molecule antioxidants and small protein antioxidants in serum samples was analyzed by measuring the total antioxidant activity (TAA) following the ABTS/H_2_O_2_/HRP method developed by Arnao^30^ with modifications by Gonzalo-Calvo^31^ (Supplementary Text S2).

### Statistical analyses

Statistical analyses were performed using SPSS (IBM Corp. Released 2020. IBM SPSS Statistics for Windows, Version 27.0. Armonk, NY: IBM Corp.) and GraphPad Prism (GraphPad Prism version 8.0.0 for Windows, GraphPad Software, San Diego, California USA, http://www.graphpad.com). The consistency of genotyping results with the Hardy–Weinberg equilibrium (HWE) was tested by exact tests (https://ihg.gsf.de/cgi-bin/hw/hwa1.pl). A two-sided P-value < 0.05 was considered statistically significant. Kolmogorov–Smirnov or Shapiro–Wilk tests were used to determine whether the set of observations followed normal distributions. Student’s *t* and Mann–Whitney U tests were used to study normally and non-normally distributed parameters, respectively. Analyses of variance (ANOVA) and Kruskal–Wallis tests were applied to study differences between three groups with normally and non-normally distributed parameters, respectively. The Wilcoxon signed-rank test was used to compare paired blood test results from baseline and follow-up. The associations between gene variants and follow-up data were tested by logistic regression analyses. Principal component analysis (PCA) was performed with R (2020, R Core Team, Vienna, Austria). The impact of the tested polymorphisms on the clinical endpoint (defined as LT or liver-related death), as well as diagnosis of HCC, was estimated with Cox regression and Kaplan–Meier survival analysis.

## Results

### Description of the study cohort

Clinical details of the study cohort are presented in Table 1. In total, 313 adults with AIH (70% women) were recruited; their median age was 36 (range 18–83) years. Pure AIH, AIH-PSC, and AIH-PBC were diagnosed in 206 (66%), 77 (25%), and 30 (9%) recruited patients, respectively. The median duration of the disease was 4 (range 0–33) years. In total, 60 (19%) patients were therapy naïve at inclusion. Liver cirrhosis, diagnosed by LB or LSM and by clinical signs of decompensation, was present in 130 (42%) patients at the enrolment in the study. Almost 70% of the entire cohort received steroid-based therapy, which is summarized in Table 1. Almost all patients with cholestatic variants were treated with ursodeoxycholic acid. Approximately 40% of patients were on steroid-based therapy after 2 years of therapy, whereas more than 20% of cases achieved long-term biochemical remission (more than 2 years) and were not receiving immunosuppression at the baseline.

**Table 1:** Clinical and biochemical data of the study cohort.

	Entire cohort	Pure AIH	AIH-PSC AIH-PBC
Total participants, n	313	206	107
Female, n (%)	218 (70%)	161 (78%)	57 (53%)
Age (years)	36 (18–84)	37 (18–84)	34 (18–68)
BMI (kg/m^2^)	23.0 (15.6–40.9)	23.6 (16.6–40.9)	22.6 (15.6–33.6)
Duration of the disease (years)	4.0 (0–37)	3.4 (0–37)	4.3 (0–27)
ALT (IUI/l, normal < 56)	54 (8–3400)	44 (8–3400)	94 (8–1233)
AST (IU/l, normal < 40)	44 (5–1543)	40 (16–1100)	72 (5–1543)
ALP (IU/l, normal < 120)	99 (23–1193)	85 (23–344)	184 (39–1193)
Bilirubin (mg/dl, normal < 1.2)	1.0 (0.2–34.1)	1.0 (0.2–34.1)	1.0 (0.2–18.6)
IgG (mg/dl, normal < 1600)	1488 (318–6508)	1475 (677–6508)	1508 (318–4381)
Platelets (G/l)	179 (21–667)	157 (21–519)	210 (21–667)
FIB-4 (points)	1.7 (0.1–37.7)	1.8 (0.2–37.7)	1.3 (0.1–14.3)
MELD (points)	8.5 (6.4–36.9)	8.5 (6.4–36.9)	7.7 (6.4–21.5)
LSM (kPa)	11.2 (4.0–74.0)	11.4 (4.0–52.4)	10.6 (4.4–74.0)
**Treatment**			
Steroid-based therapy	217 (69%)	138 (64%)	79 (74%)
AZA monotherapy	21 (7%)	18 (8%)	3 (3%)
Other treatment or none	97 (31%)	59 (27%)	38 (36%)

*AIH* autoimmune hepatitis, *ALP* alkaline phosphatase, *ALT* alanine aminotransferase, *AST* aspartate aminotransferase, *AZA* azathioprine, *FIB-4* Fibrosis-4, *IgG* immunoglobulin G, *kPa* kilopascal, *LSM* liver stiffness measurement, *MELD* model of end-stage liver disease, *PBC* primary biliary cholangitis, *PLT* platelet count, *PSC* primary sclerosing cholangitis. Values are expressed as medians (ranges), unless stated otherwise.

### *MARC1* shows protective effects in patients with AIH

The five genetic variants were successfully genotyped in all patients. Table 2 presents the distributions of genotypes. All variants were within the Hardy–Weinberg equilibrium, underscoring robust genotyping.

**Table 2:** Genotype frequencies in the study group.

	*MARC1*	*HSD17B13*	*PNPLA3*	*TM6SF2*	*MBOAT7*
**Entire cohort (n = 313)**					
Wild-type	164 (52.4%)	165 (52.7%)	188 (60.1%)	262 (83.7%)	108 (34.5%)
Heterozygous variant	119 (38.0%)	119 (38.0%)	109 (34.8%)	51 (16.3%)	137 (43.8%)
Homozygous variant	30 (9.6%)	29 (9.3%)	16 (5.1%)	0 (0%)	68 (21.7%)
**Patients with AIH without cholestatic overlaps (n = 206)**					
Wild-type	110 (53.4%)	106 (51.4%)	129 (62.6%)	169 (82.0%)	71 (34.5%)
Heterozygous variant	76 (36.9%)	77 (37.4%)	66 (32.0%)	37 (18.0%)	86 (41.7%)
Homozygous variant	20 (9.7%)	23 (11.2%)	11 (5.4%)	0 (0%)	49 (23.8%)
**Patients with cholestatic overlaps of AIH: AIH-PSC or AIH-PBC (n = 107)**					
Wild-type	54 (50.5%)	59 (55.1%)	59 (55.1%)	93 (86.9%)	37 (34.6%)
Heterozygous variant	43 (40.2%)	42 (39.3%)	43 (40.2%)	14 (13.1%)	51 (47.7%)
Homozygous variant	10 (9.3%)	6 (5.6%)	5 (4.7%)	0 (0%)	19 (17.7%)

*HSD17B13* hydroxysteroid 17β-dehydrogenase 13, *MARC1*, mitochondrial amidoxime-reducing component 1, *MBOAT7* membrane-bound O-acyltransferase domain containing 7, *PNPLA3* patatin-like phospholipase domain-containing protein 3, *TM6SF2* transmembrane 6 superfamily member 2.

**Figure 1:**
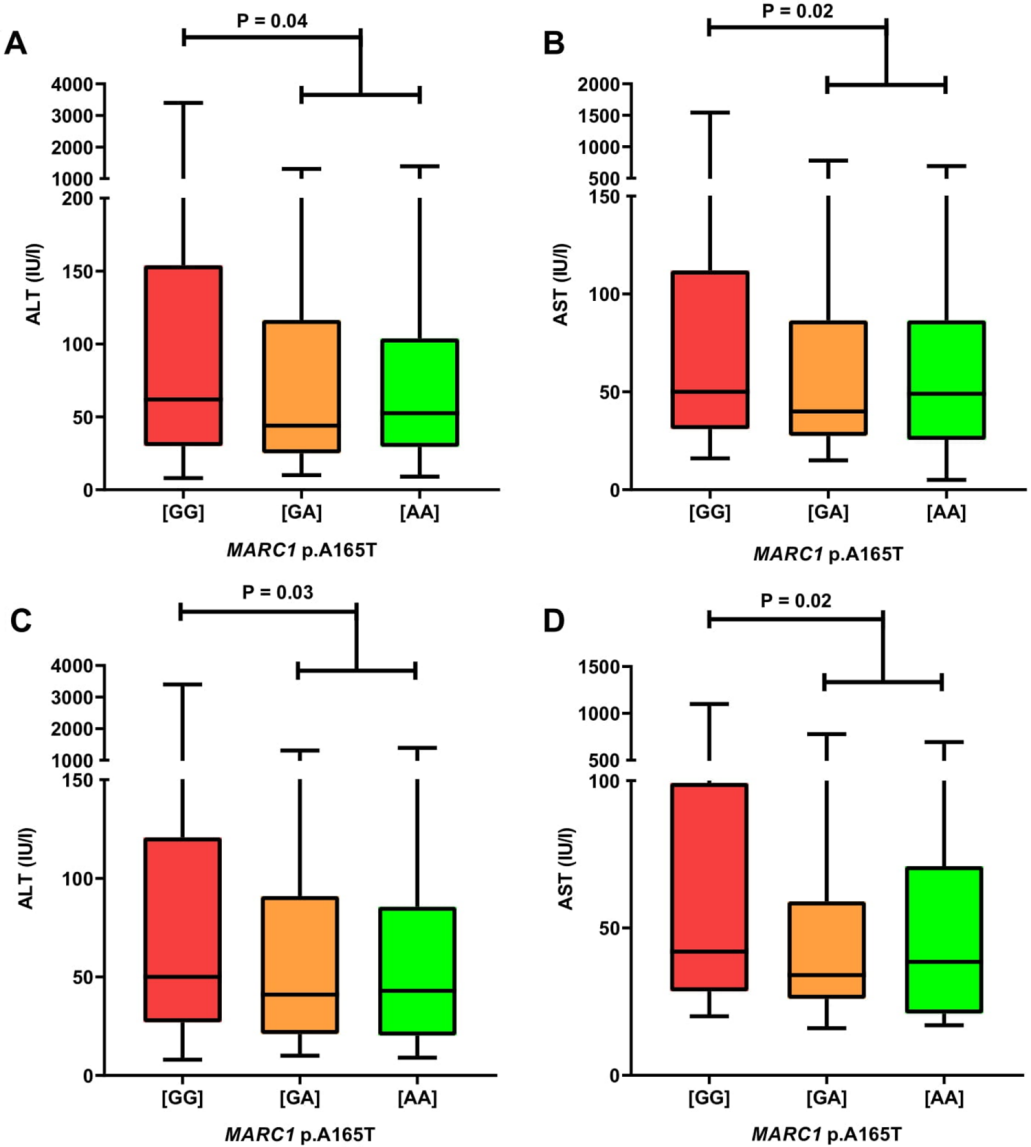
*MARC1* minor allele is associated with lower serum ALT and AST activities in patients with AIH (**A**,**B** results in the entire study cohort, n = 313; **C**,**D** results in patients with pure AIH (i.e. without cholestatic overlaps), n = 206). [GG], *MARC1* wild-type; [GA], *MARC1* heterozygous variant; [AA], *MARC1* homozygous variant; ALT, serum alanine aminotransferase level; AST, aspartate aminotransferase; *MARC1*, mitochondrial amidoxime-reducing component 1.

The *MARC1* polymorphism showed prominent modulating effects on the AIH phenotype. In the entire cohort of 313 individuals, carriers of the *MARC1* minor allele had significantly lower serum activities of ALT (P = 0.04) and AST (P = 0.02) (Fig. 1A,B) at baseline. Comparable results were found in patients with pure AIH (n = 206); carriers of at least one minor allele had significantly lower serum ALT (P = 0.03) and AST activities (P = 0.02) (Fig. 1C,D).

**Figure 2:**
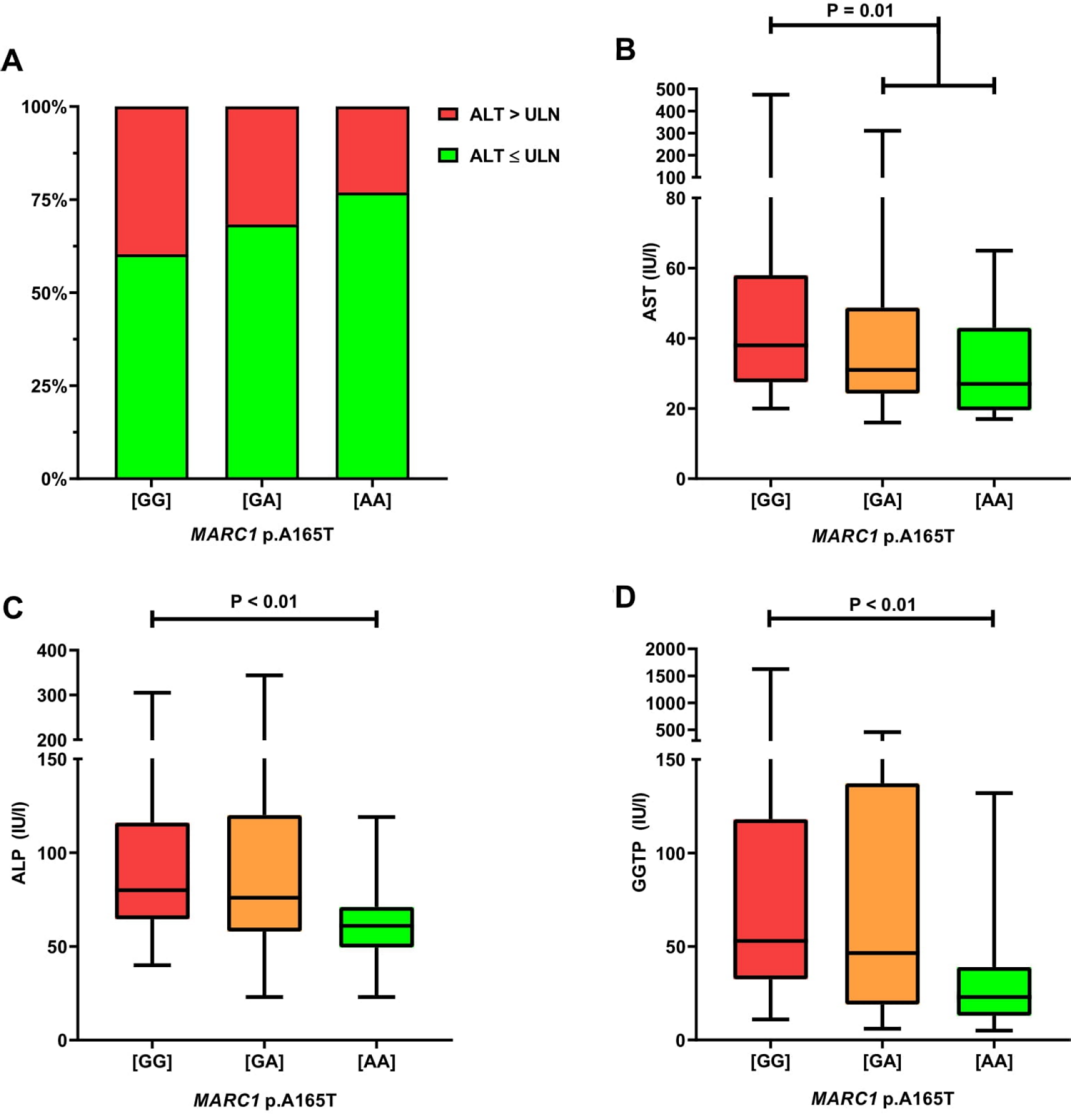
*MARC1* variant and clinical data in patients treated for at least 6 months for AIH without cholestatic overlaps (n = 146; **A** distribution of patients with normal or increased ALT in relation to the *MARC1* genotype; **B-D** effects of this variant on serum AST, ALP and GGT). [GG], *MARC1* wild-type; [GA], *MARC1* heterozygous variant; [AA], *MARC1* homozygous variant; ALP, alkaline phosphatase; ALT, alanine aminotransferase; AST, aspartate aminotransferase; GGT, gamma-glutamyl transferase; UNL, upper normal limit; *MARC1*, mitochondrial amidoxime-reducing component 1.

Figure 2 shows the results of 146 patients with pure AIH who were treated for ≥ 6 months. In these patients, the frequency of individuals with normal ALT increased with the number of protective *MARC1* alleles (Fig. 2A). As shown in Fig. 2B, carriers of the protective *MARC1* allele had significantly lower serum AST activities (P = 0.01), and homozygous carriers of the protective allele presented with lower ALP (P < 0.01, Fig. 2C) and GGT activities (P < 0.01, Fig. 2D) as compared to patients with the wild-type genotype. Carriers of the *MARC1* minor allele also had lower APRI (median 0.61, range 0.15–7.28) compared to the carriers of the wild-type genotype (median 0.81, range 0.18–14.04; P = 0.02). Finally, the *MARC1* p.A165T polymorphism was associated with lower immunoglobulin G (IgG) concentrations (P < 0.01) among AIH patients with elevated IgG levels (> 1600 mg/dL; n = 53). LSM, MELD, and liver histology were not modulated by this polymorphism.

Among patients who were followed up for 12 months (data available for 145 patients), carriers of at least one *MARC1* p.A165T allele showed lower ALT and AST activities at baseline (both P < 0.05) compared to carriers of the wild-type genotype (Supplementary Table S2). Moreover, the presence of at least one *MARC1* allele was also associated with a trend (P = 0.05) for improved FIB-4 during follow-up (Supplementary Table S2).

The results of the genotype–phenotype association analysis in a subgroup of patients with AIH-PSC and AIH-PBC are presented in Supplementary Text S3 and Fig. S1.

### *PNPLA3* variant is associated with a higher MELD score, whereas the *MBOAT7* polymorphism might modulate HCC risk

**Figure 3:**
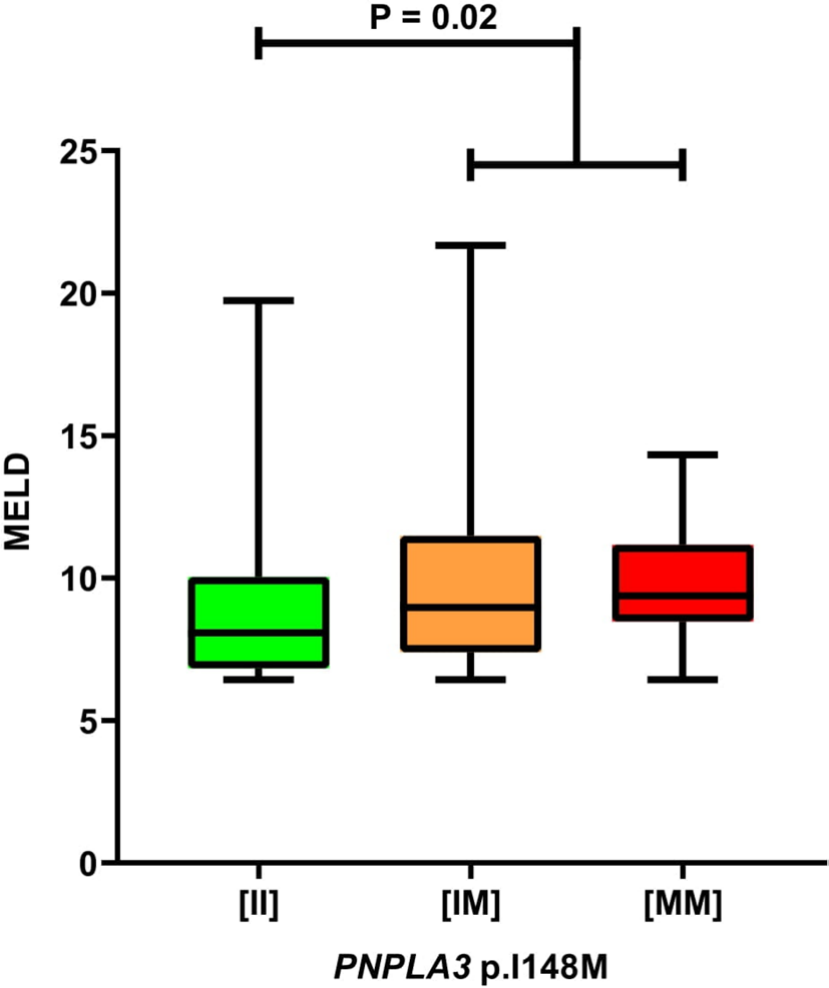
*PNPLA3* p.I148M and MELD score in patients with AIH without cholestatic overlaps treated for at least 6 months (n = 146). [II], *PNPLA3* wild-type; [IM] *PNPLA3* heterozygous variant; [MM], *PNPLA3* homozygous variant; MELD, model of end-stage liver disease; *PNPLA3***,** patatin-like phospholipase domain-containing protein 3.

A significantly higher MELD score (P = 0.02, Fig. 3) among patients with pure AIH treated ≥ 6 months (n = 146) who carried at least one *PNPLA3* p.148M risk allele was the only association between patients’ characteristics and the *PNPLA3* variant at baseline. Regardless of increasing MELD, the presence of this allele did not increase the risk of transplantation or death (1.03, 95% CI 0.73–1.46, P = 0.86) and did not affect survival in Kaplan–Meier analysis. During the 12-month follow-up, patients with the *PNPLA3* wild-type genotype showed a significant (P = 0.03) decrease in serum ALT activity, whereas a trend to increased FIB-4 during the follow-up period was observed in carriers of at least one *PNPLA3* p.148M risk allele (P = 0.06, Supplementary Table S3).

*HSD17B13*, *TM6SF2,* and *MBOAT7* variants were not associated with liver function tests, LSM, FIB-4, APRI, or MELD (all P > 0.05). Moreover, none of the tested polymorphisms was associated with prolonged steroid-based treatment (i.e., ≥ 2 years) or cirrhosis at diagnosis. None of the five studied genetic variants was associated with LT or death (all P > 0.05) in our cohort.

HCC was diagnosed in eight patients, all with cirrhosis. Interestingly, we found an association between *MBOAT7* and HCC. All but one patient diagnosed with HCC carried at least one *MBOAT7* minor allele [T]. Accordingly, the *MBOAT7* [TT] genotype was associated with an increased risk of developing HCC (odds ratio (OR) 3.71, 95% CI 1.22–11.28, P = 0.02). No other evaluated polymorphisms were found to modify the risk of HCC in our study.

### Presence of the protective *MARC1* variant is associated with increased antioxidant capacity

**Figure 4:**
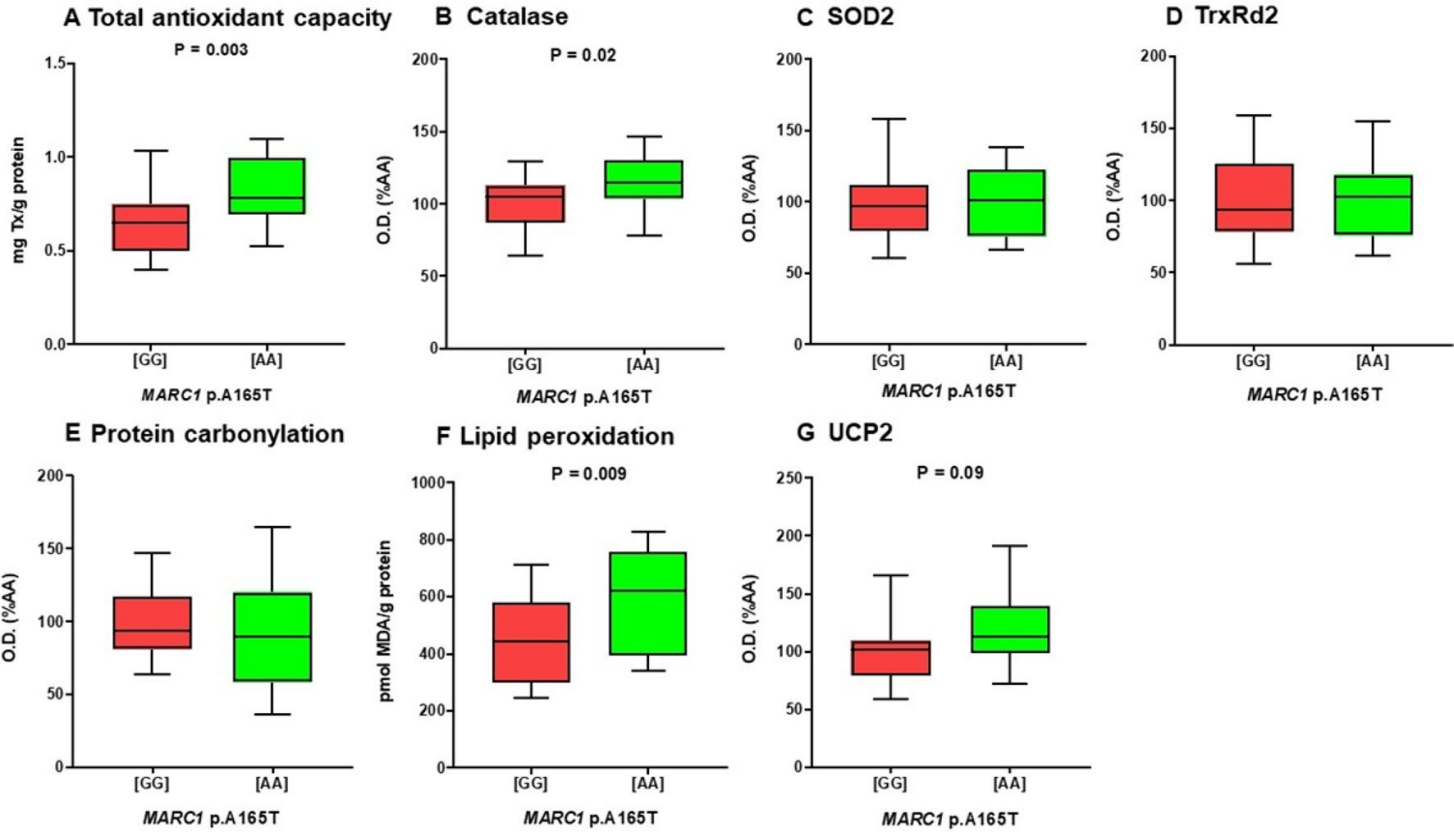
The status of antioxidant defence and oxidative stress manifestation in serum samples of AIH patients in relation to the p.A165T *MARC1* genotype. Analysis was performed in randomly selected 25 patients carrying the *MARC1* wild-type genotype and 14 *MARC1* homozygous carriers of the protective allele. [GG], *MARC1* wild-type; [AA], *MARC1* homozygous variant; *MARC1*, mitochondrial amidoxime-reducing component 1; SOD2, superoxide dismutase 2; TrxRd2, thioredoxin reductase 2; UCP2, uncoupling protein 2.

Based on the above results that linked the *MARC1* polymorphism to liver injury biomarkers, we performed further evaluation. We randomly selected 25 individuals carrying the *MARC1* wild-type genotype [GG] and 14 *MARC1* homozygous carriers of the protective allele [AA]. Supplementary Table S4 summarizes the characteristics of these patients. Both groups of patients did not differ significantly in terms of clinical characteristics, apart from lower ALP among carriers of the protective variant. As reflected by PCA (Supplementary Fig. S2), the stages of liver disease (i.e., FIB-4, LSM, and MELD) did not differ between carriers of the *MARC1* genotypes. The TAA in serum, which represents small-molecule and protein antioxidants, was higher in patients carrying the *MARC1* variant compared to the wild-type genotype (P = 0.003, Fig. 4A). Moreover, the catalase levels in carriers of the *MARC1* protective allele were also increased (P = 0.02, Fig. 4B; Supplementary Fig. S3). On the other hand, the levels of SOD2 and TrxRd2, two antioxidant enzymes involved in the mitochondrial antioxidant defense system, did not differ (Fig. 4C,D; Supplementary Fig. S3). Interestingly, the higher antioxidant capacity (TAA) and higher levels of catalase observed in *MARC1* homozygotes correlated with an increase in oxidatively damaged lipids (peroxidation), while the level of carbonylated proteins remained unchanged (Fig. 4E,F; Supplementary Fig. S4). The level of UCP2 was similar in serum samples from carriers of the *MARC1* variant and patients carrying the *MARC1* wild-type genotype (P > 0.05, Fig. 4G; Supplementary Fig. S3). This finding is supported by above-described observation that the levels of other mitochondrial proteins (SOD2 and TrxRd2), associated with the antioxidant response and localized in the mitochondrial matrix, did not change either similarly to UCP2 (Fig. 4C,D; Supplementary Fig. S3).

## Discussion

In this study, we aimed to investigate the role of genetic predisposition as a modulator of AIH. By analyzing an extensive cohort of patients, we detected lower indices of liver injury in the carriers of the *MARC1* p.A165T polymorphism. This observation was coupled with the improved marker of liver fibrosis, APRI, in patients treated for AIH for at least 6 months. Analyses of patients blood samples demonstrated that the effects of the *MARC1* polymorphism might be related to increased antioxidant capacity in the sera of patients carrying the protective genotype. Moreover, we observed that *PNPLA3* p.I148M was associated with higher MELD and that the *MBOAT7* polymorphism was linked to an increased risk of developing HCC in the course of AIH.

The presence of the inherited modifiers of AIH was previously suspected^32^. Since AIH encompasses a heterogeneous group of patients, we decided to analyze both the entire cohort and as well as separate subgroups of patients based on the duration of therapy and the presence of cholestatic variants of AIH. By analyzing the entire group of 313 patients, we detected lower serum transaminase activities among patients carrying the *MARC1* polymorphism. This association was also apparent in individuals that were treated for AIH for at least 6 months: they have lower transaminases and lower APRI score. The effect of the *MARC1* polymorphism on liver fibrosis marker was maintained during the 12-month follow-up. On the other hand, we did not detect any significant effect of *MARC1* on the results of liver biopsies. It has to be kept in mind that not all patients in our cohort received liver biopsies at baseline. Patients with already established diagnoses (confirmed by previously performed liver biopsy before referral to our center) and during maintenance therapy did not undergo liver biopsy at inclusion. This relatively low number of liver biopsies lowered the power of this analysis. Overall, we believe that although the presence of the *MARC1* minor allele is associated with decreased serum markers of liver injury, this polymorphism has only limited effects on the progression of liver fibrosis. This is also underscored by the lack of association between this variant and LSM.

*MARC1* is an enzyme attached to the mitochondrial membrane, a molybdenum-containing nitric oxide synthase that reduces the physiological substrate *N*(omega)-hydroxy-l-arginine. Luukkonnen et al. showed that the minor allele might increase intrahepatic phosphatidylcholines^9^. The missense variant p.A165T in *MARC1* was previously linked to protection against cirrhosis, lower hepatic fat contents, and lower risk of developing fatty liver^8^. In our cohort, the *MARC1* protective variant correlated with increased antioxidant capacity, manifested as an increased level of catalase (ubiquitous primary antioxidant enzyme) and increased total antioxidant activity in serum. The increased antioxidant capacity can also be considered as a response to counteract augmented oxidative stress, which is mirrored by higher lipid peroxidation in *MARC1* homozygotes. Interestingly, the *MARC1* genotype seemed to be the main driver of changes in antioxidant capacity since other clinical characteristics, apart from lower ALP, did not differ between patients included in this analysis (Supplementary Table 4). The unchanged level of mitochondrial markers, reported in the literature to be present in the serum of the patients with severe liver damage^33,34^, supports the assumption that increased antioxidant capacity can play a protective role in patients carrying the protective *MARC1* polymorphism.

The genetic variants that we chose to genotype in our patients with AIH were previously shown to modify liver fibrosis and/or steatosis in patients with viral, alcoholic, and non-alcoholic fatty liver disease^3,35,36^. To date, studies concerning the role of genetics on AIH have mainly focused on the associations with human leukocyte antigen^13–15^. In a recent analysis, the common *PNPLA3* p.I148M polymorphism was associated with the progression of AIH^22^ and clinical endpoints, defined as LT or death^22^. In our study, we were not able to replicate any of these findings; however, we found an association between the *PNPLA3* variant and higher MELD. Survival analyses of our patients disclosed no correlation between liver transplantation or death and the studied variants (of note, 96 (28%) patients reached these endpoints). The differences between the findings of our study and those of a previous publication^22^ underscore the heterogeneity of patients with AIH and the different diagnostic and therapeutic strategies established in each center. Our center serves as a referral unit for patients with AIH in Poland, which might be one of the reasons why our cohort was characterized by a high prevalence of AIH-PSC and AIH-PBC overlaps with a relatively high number of patients with cirrhosis. These variants of AIH are typically more difficult to treat, with a wide variation in clinical decisions together with these patients, even in referral centers^37^.

We detected a potential link between the *MBOAT7* polymorphism and the risk of developing HCC. In our study group, eight patients had HCC, which is in line with other reports that indicated a relatively low prevalence of HCC in AIH^1^. *MBOAT7* was previously identified as a risk factor of HCC^38^, and we report for the first time this association in patients with AIH. However, given the low incidence of this tumor in autoimmune liver diseases, replication in a larger cohort is necessary to confirm this result.

In conclusion, in our candidate gene study, we found potential protective effects of the *MARC1* polymorphism in patients with AIH. The *PNPLA3* and *MBOAT7* variants might be associated with a more severe AIH. These results allude to the role of inherited predisposition in the course of AIH. Analyses of intracellular processes that are affected by the studied variants might provide new functional insights into the pathogenesis of AIH.

## Supplementary Information

Supplementary Information 1.

Supplementary Information 2.

## Abbreviations

AIH: Autoimmune hepatitis
ALP: Alkaline phosphatase
ALT: Alanine aminotransferase
APRI: Aspartate aminotransferase to platelet ratio index
AST: Aspartate aminotransferase
CAT: Catalase
FIB-4: Fibrosis-4
GWAS: Genome-wide association study
HLA: Human leukocyte antigen
HCC: Hepatocellular carcinoma
HSD17B13: Hydroxysteroid 17β-dehydrogenase 13
IgG: Immunoglobulin G
LB: Liver biopsy
LSM: Liver stiffness measurement
LT: Liver transplantation
UNL: Upper normal limit
MARC1: Mitochondrial amidoxime-reducing component 1
MBOAT7: Membrane-bound O-acyltransferase domain containing 7
MELD: Model of end-stage liver disease
NAFLD: Non-alcoholic fatty liver disease
PBC: Primary biliary cholangitis
PLT: Platelet count
PNPLA3: Patatin-like phospholipase domain-containing protein 3
PSC: Primary sclerosing cholangitis
SOD2: Superoxide dismutase 2
TAA: Total antioxidant activity
TM6SF2: Transmembrane 6 superfamily member 2
TrxRd2: Thioredoxin reductase 2
UCP2: Uncoupling protein 2
