# Supplementary material for: MARC1 p.A165T variant is associated with decreased markers of liver injury and enhanced antioxidant capacity in autoimmune hepatitis
Source: Sci Rep. 2021 Dec 23;11:24407. doi: 10.1038/s41598-021-03521-3 (PMC8702547; doi:10.1038/s41598-021-03521-3)
Supplement: Supplementary file 1 — Supplementary Information 1. [file 41598_2021_3521_MOESM1_ESM.docx]

**Supplementary Table S1.** Assays that were used for genotyping.

| **SNP rsID** | **Gene** | **Assay Number** |
| --- | --- | --- |
| rs2642438 | *MARC1* | C___1271599_10 |
| rs738409 | *PNPLA3* | C______7241_10 |
| rs58542926 | *TM6SF2* | C__89463510_10 |
| rs641738 | *MBOAT7* | C___8716820_10 |
| rs72613567 | *HSD17B13* | Custom assay |

**Supplementary Table S2.** The changes during 12 months follow-up of the patients with pure AIH (available data in 145 patients), divided as carriers of the one or two copies allele of *MARC1* p.A165T.

|  |  | Baseline | Follow-up  12 months | P-value |
| --- | --- | --- | --- | --- |
| *MARC1*  p.A165T [GG]  (n = 76) | \| ALT (IU/l) \| \| --- \| | 43* | 31 | 0.01 |
|  | \| AST (IU/l) \| \| --- \| | 37* | 34 | 0.20 |
|  | \| Bilirubin (mg/dl) \| \| --- \| | 0.8 | 0.8 | 0.42 |
|  | \| Creatinine (mg/dl) \| \| --- \| | 0.7 | 0.8 | 0.08 |
|  | \| MELD \| \| --- \| | 7.5 | 7.5 | 0.95 |
|  | \| FIB-4 \| \| --- \| | 1.68 | 1.59 | 0.83 |
| *MARC1* p.A165T [GA] + [AA]  (n = 69) | \| ALT (IU/l) \| \| --- \| | 31* | 28 | 0.83 |
|  | \| AST (IU/l) \| \| --- \| | 29* | 29 | 0.77 |
|  | \| Bilirubin (mg/dl) \| \| --- \| | 0.9 | 0.7 | 0.18 |
|  | \| Creatinine (mg/dl) \| \| --- \| | 0.7 | 0.7 | 0.43 |
|  | \| MELD \| \| --- \| | 8.4 | 8.5 | 0.43 |
|  | \| FIB-4 \| \| --- \| | 1.36 | 1.29 | 0.05 |

Values are expressed as medians. The data tested were not normally distributed (confirmed by Shapiro-Wilk test), thus the Wilcoxon signed-rank test was used to compare between baseline and follow-up blood test. The p-value < 0.05 considered significant. * - significant differences between groups at baseline (both P < 0.05) evaluated by U Mann Whitney test.

**Supplementary Table S3.** The changes during 12 months follow-up of the patients with pure AIH (available data in 145 patients), divided as carriers of the one or two copies allele of *PNPLA3* p.I148M.

|  |  | **Baseline** | **Follow-up**  **12 months** | **P-value** |
| --- | --- | --- | --- | --- |
| ***PNPLA3* p.i148m**  **[IM] + [MM]**  **(n = 51)** | \| ALT (IU/l) \| \| --- \| | 51.0 | 44.2 | 0.81 |
|  | \| AST (IU/l) \| \| --- \| | 46.3 | 45.6 | 0.48 |
|  | \| Bilirubin (mg/dl) \| \| --- \| | 1.1 | 1.1 | 0.29 |
|  | \| Creatinine (mg/dl) \| \| --- \| | 0.7 | 0.8 | 0.15 |
|  | \| MELD \| \| --- \| | 9.1 | 8.8 | 0.80 |
|  | \| FIB-4 \| \| --- \| | 2.46 | 2.94 | 0.06 |
| ***PNPLA3***  **p.I148M**  **[II]**  **(n = 94)** | \| ALT (IU/l) \| \| --- \| | 64.7 | 44.2 | 0.03 |
|  | \| AST (IU/l) \| \| --- \| | 49.0 | 40.7 | 0.18 |
|  | \| Bilirubin (mg/dl) \| \| --- \| | 1.0 | 1.0 | 0.26 |
|  | \| Creatinine (mg/dl) \| \| --- \| | 0.8 | 0.8 | 0.85 |
|  | \| MELD \| \| --- \| | 8.5 | 8.8 | 0.36 |
|  | \| FIB-4 \| \| --- \| | 2.15 | 2.17 | 0.94 |

Values are expressed as medians. The data tested were not normally distributed (confirmed by Shapiro-Wilk test), thus the Wilcoxon signed-rank test was used to compare between baseline and follow-up blood test. The p-value < 0.05 considered significant.

**Supplementary Table S4.** Clinical characterization of the randomly selected patients evaluated for oxidative stress and antioxidant defence status in respect to *MARC1* genotype.

|  | ***MARC1* p.A165T  [GG]** | ***MARC1***  **p.A165T**  **[AA]** | **P-value** |
| --- | --- | --- | --- |
| **Total participants, n** | 25 | 14 | - |
| **Female, n (%)** | 16 (67%) | 13 (93%) | <0.01 |
| **Age (years)** | 39 (18-67) | 34 (18-57) | 0.22 |
| **BMI (kg/m2)** | 21.7  (16.6-34.2) | 23.8  (20.3-30.8) | 0.18 |
| **Duration of the disease (years)** | 5.8 (1-27) | 4.6 (0-26) | 0.24 |
| **ALT (IU/l, normal <56)** | 38 (12-895) | 35 (9-1393) | 0.66 |
| **AST (IU/l, normal <40)** | 38 (21-1100) | 28 (17-694) | 0.26 |
| **ALP (IU/l, normal <120)** | 85 (45-305) | 63 (23-186) | <0.01 |
| **Bilirubin (mg/dl, normal <1.2)** | 1.0 (0.3-6.8) | 1.1 (0.4-1.9) | 0.71 |
| **IgG (mg/dl, normal <1600)** | 1466  (768-3476) | 1305  (677-2653) | 0.32 |
| **Platelets (G/l)** | 158 (21-416) | 109 (27-352) | 0.72 |
| **FIB-4 (points)** | 1.9 (0.4-11) | 2.8 (0.3-6) | 0.87 |
| **MELD (points)** | 9.3 (6.4-22) | 9.0 (6.4-18) | 0.89 |
| **LSM (kPa)** | 11.1 (5.0-45.3) | 11.1 (4.8-40.3) | 0.87 |

Abbreviations: see Table 2. Values are presented as medians (ranges). The U Mann-Whitney test and chi2 test were used to compare between the subgroups.

**Supplementary Figure S1.** Association between the *HSD17B13* variant and lower serum GGT in patients with AIH-PSC and AIH-PBC.

**Abbreviations:** [TT], *HSD17B13* wild-type [TAT], *HSD17B13* heterozygous variant; [TATA], *HSD17B13* homozygous variant; GGT, gamma-glutamyl transferase.

**Supplementary Figure S2.** Principal component analysis of clinical data in randomly selected patients evaluated for oxidative stress and antioxidant defence status in relation to the *MARC1* genotype.


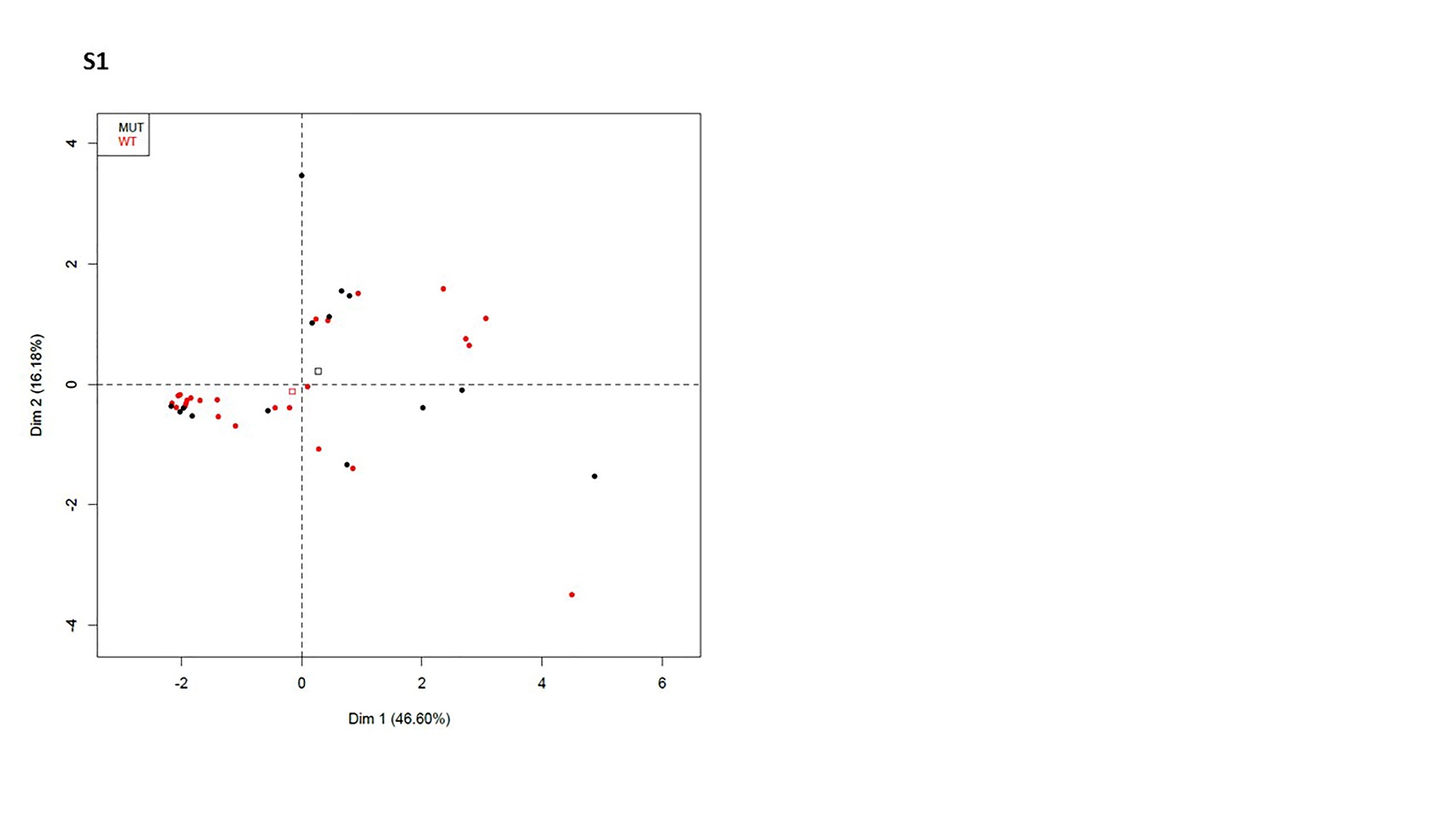


**Supplementary Figure S3.** Representative Western blot images of A) catalase, B) superoxide dismutase 2 (SOD2), C) thioredoxin reductase 2 (TrxRd2) and D) uncoupling protein 2 (UCP2) presented in the Figure 4 (B, C, D, G). Revert staining was used to determine the total protein - loading control E) for catalase and UCP2 and F) for SOD2 and TrxRd2. Original images of the Western blot are presented in Supplementary Materials – original images.


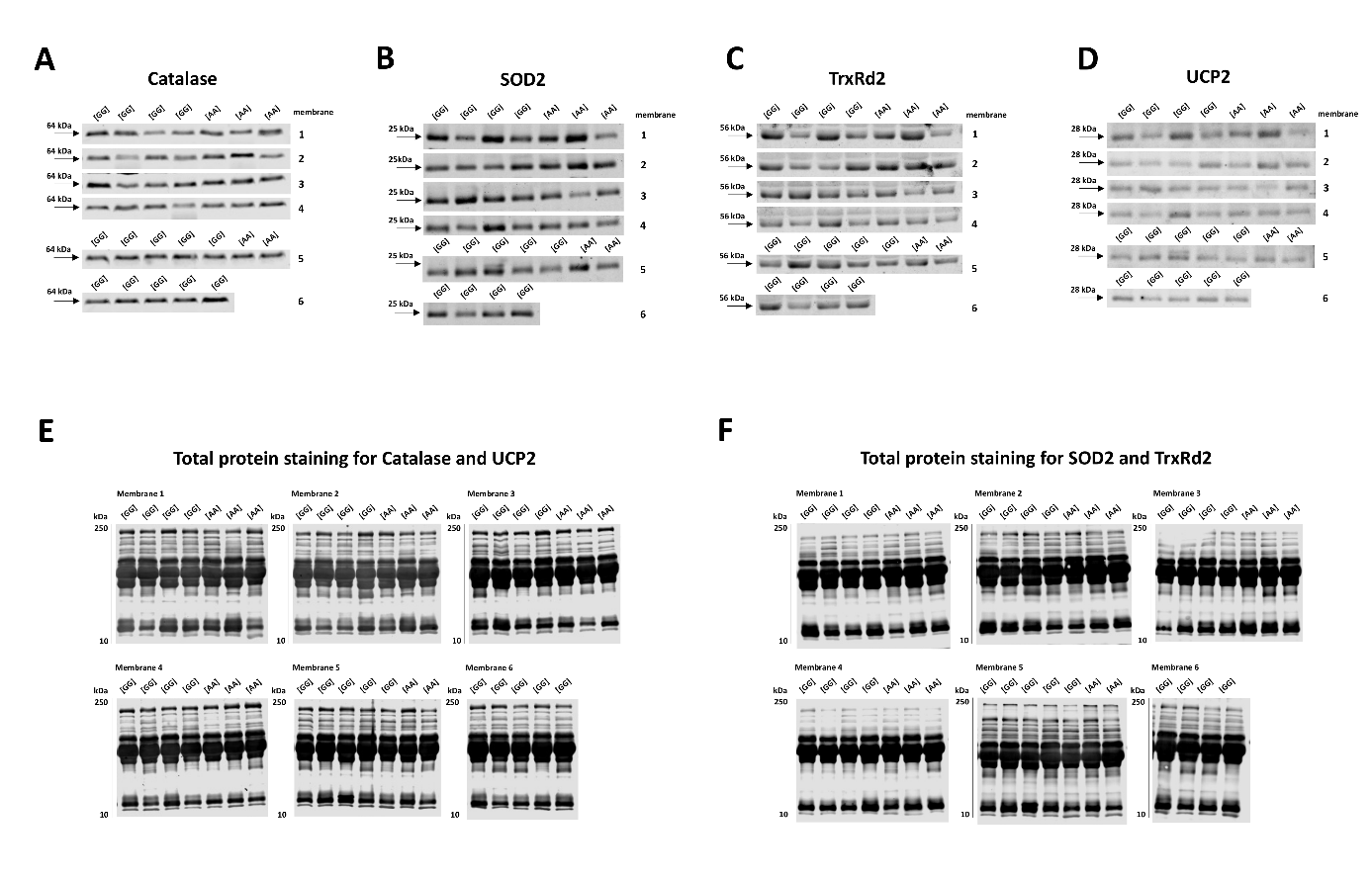


**Supplementary Figure S4.** Representative Western blot images of protein carbonylation (DNP) presented in the Figure 4E. Revert staining was used to determine the total protein – loading control.


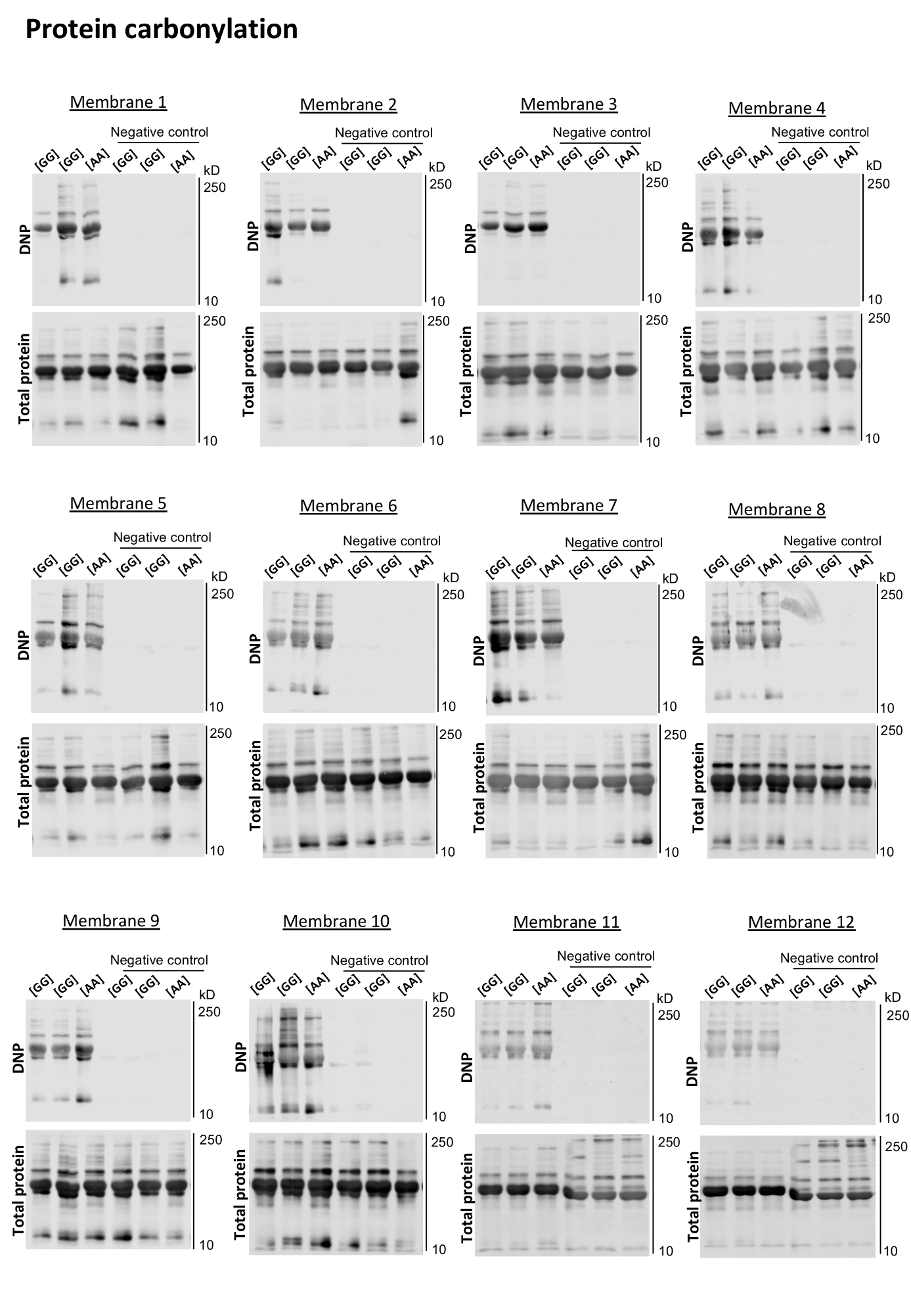


**SUPPLEMENTARY TEXT 1**

**Genotyping**

For genotyping of rs2642438 (*MARC1*), rs738409 (*PNPLA3*), rs58542926 (*TM6SF2*) and rs641738 (*MBOAT7*) commercially available assays were used (ThermoFisher Scientific, Supplementary Table S5). For rs72613567 (*HSD17B13*), the flanking sequence of the SNP was supplied to ThermoFisher Scientific and a custom assay was designed by the company. Genotyping was performed as follows: In brief, DNA was mixed with genotyping master mix (comprising polymerase, nucleotides and buffer) and genotyping assay (containing primers and probes). The PCR reaction was run on a TaqMan 7500 Fast System using the following protocol: 25°C for 1 min (pre-read), 40 cycles of 95°C for 20 sec and 60°C for 30 sec. The fluorescent signal was detected during the post read period at 25°C for 1min. Data were analyzed using SDS v2.3 software.

**SUPPLEMENTARY TEXT 2**

**Western Blot Analysis**

Serum samples were treated with RIPA buffer (Sigma-Aldrich R0278) for 20 minutes and after centrifuged for 15 minutes at 16,000 ×g. After centrifugation, the supernatants were transferred into fresh tubes. The protein concentration in the serum lysates was estimated using Bradford’s method. Then, the samples were reduced by adding Laemmli buffer and heating for 5 minutes at 95 °C. We separated 50 µg of protein of each lysate by SDS-PAGE on a 10% gel. Separated proteins were transferred to a LF-PVDF membrane (BioRad), which was then blocked with EveryBlot Blocking Buffer (BioRad) for 1 hour. Afterward, the following primary antibodies were added to the blocking buffer supplemented with 0.1% Tween: mouse monoclonal anti-catalase, concentrated 1:1000 (Santa Cruz Biotechnology); rabbit polyclonal anti-UCP2, 1:1000 (Invitrogen PA5-80203); mouse monoclonal anti-SOD2, 1:1000 (Santa Cruz Biotechnology sc-133134); and anti-thioredoxin reductase 2 (TrxRd2), 1:500 (Santa Cruz Biotechnology sc-166259), and incubated overnight at 4 °C. After incubation with primary antibodies, membranes were washed 3 times with TBS-Tween (0.1%) and incubated with an appropriate fluorescent secondary antibody (Li-Cor) for 1 hour at room temperature. Afterward, the membranes were washed 3 times in TBS-Tween (0.1%); once in TBS, they were scanned with the use of the Odyssey® Imaging System (Li-Cor). The signal was quantified using Image Studio Lite software (Li-Cor). The results were normalized to the signal from the total protein membrane staining obtained with the use of the Revert™ 700 Total Protein Stain Kit (Li-Cor).

**Determination of Oxidative Damage in Serum**

For lipid peroxidation (LPO) assay serum, samples were treated with thiobarbituric acid (TBA) according to manufacturer’s protocol and the MDA-TBA adducts were quantified colorimetrically by assessing the optical density at 532 nm. The results are expressed as pmol MDA/g protein.

For evaluation of protein oxidative damage, carbonyl groups in serum samples (20 μg) were derivatized to 2,4-dinitrophenol (DNP)-hydrazone by reaction with 2,4-dinitrophenylhydrazin (DNPH) following the manufacturer’s instructions. DNP-derivatized proteins were separated by electrophoresis in SDS polyacrylamide 10% gel followed by Western blotting. Revert staining (ab178020, Abcam) was used to normalize the levels of carbonyl groups.

**Serum Total Antioxidant Activity**

Total antioxidant activity was evaluated colorimetrically at 730 nm. Data are expressed as equivalents of mg Trolox/mg protein.

**SUPPLEMENTARY TEXT 3**

**Results in AIH-PSC and AIH-PBC Overlaps**

None of the studied variants had major effects on the clinical phenotypes in patients with cholestatic variants of AIH. Except for the finding that carriers of the *HSD17B13* variant presented with significantly lower serum GGT (P = 0.02, Supplementary Figure S1), we did not detect any significant effects of these variants in blood tests, non-invasive markers of liver fibrosis, MELD, or clinical outcome.
