# Supplementary material for: MARC1 p.A165T variant is associated with decreased markers of liver injury and enhanced antioxidant capacity in autoimmune hepatitis
Source: Sci Rep. 2021 Dec 23;11:24407. doi: 10.1038/s41598-021-03521-3 (PMC8702547; doi:10.1038/s41598-021-03521-3)
Supplement: Supplementary file 2 — Supplementary Information 2. [file 41598_2021_3521_MOESM2_ESM.docx]

**
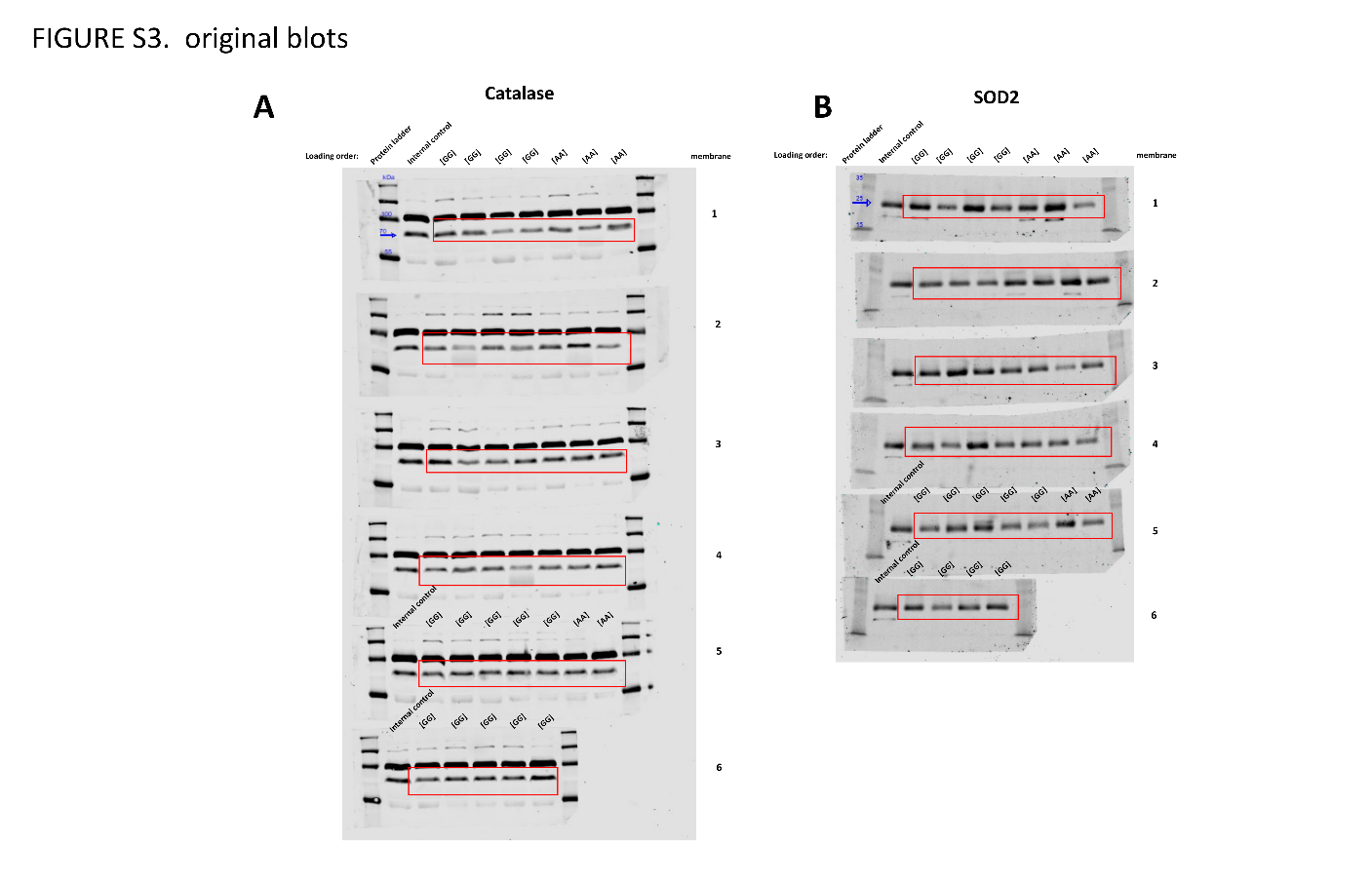
Supplementary Materials – original images related to Supplementary Figure S3.** Expanded data as original images related to Supplementary Figure S3 and A) catalase, B) superoxide dismutase 2 (SOD2), C) thioredoxin reductase 2 (TrxRd2) and D) uncoupling protein 2 (UCP2) presented in the Figure 4 (B, C, D, G). Revert staining was used to determine the total protein - loading control E) for catalase and UCP2 and F) for SOD2 and TrxRd2. The red border indicated the area used to create the Supplementary Figure S3.

**
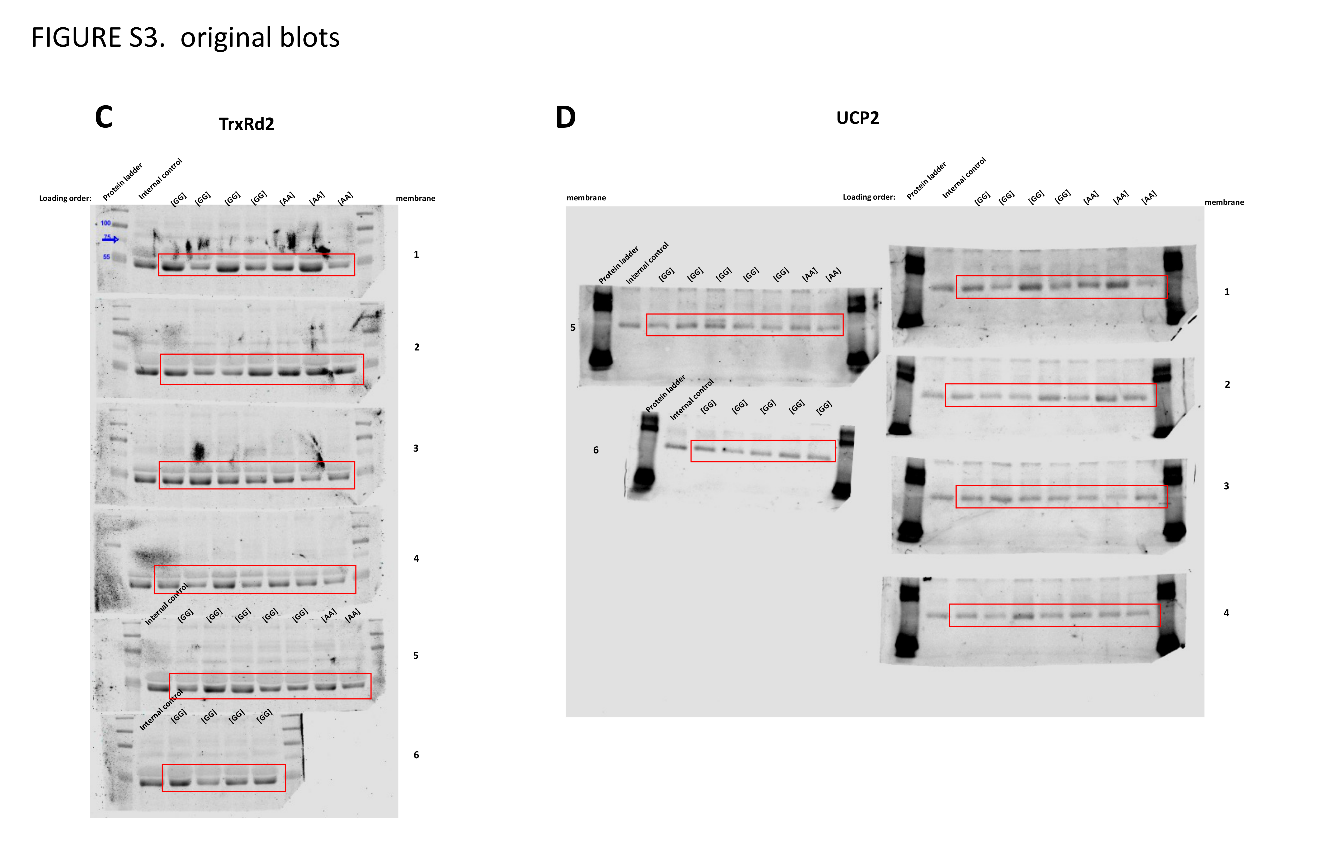

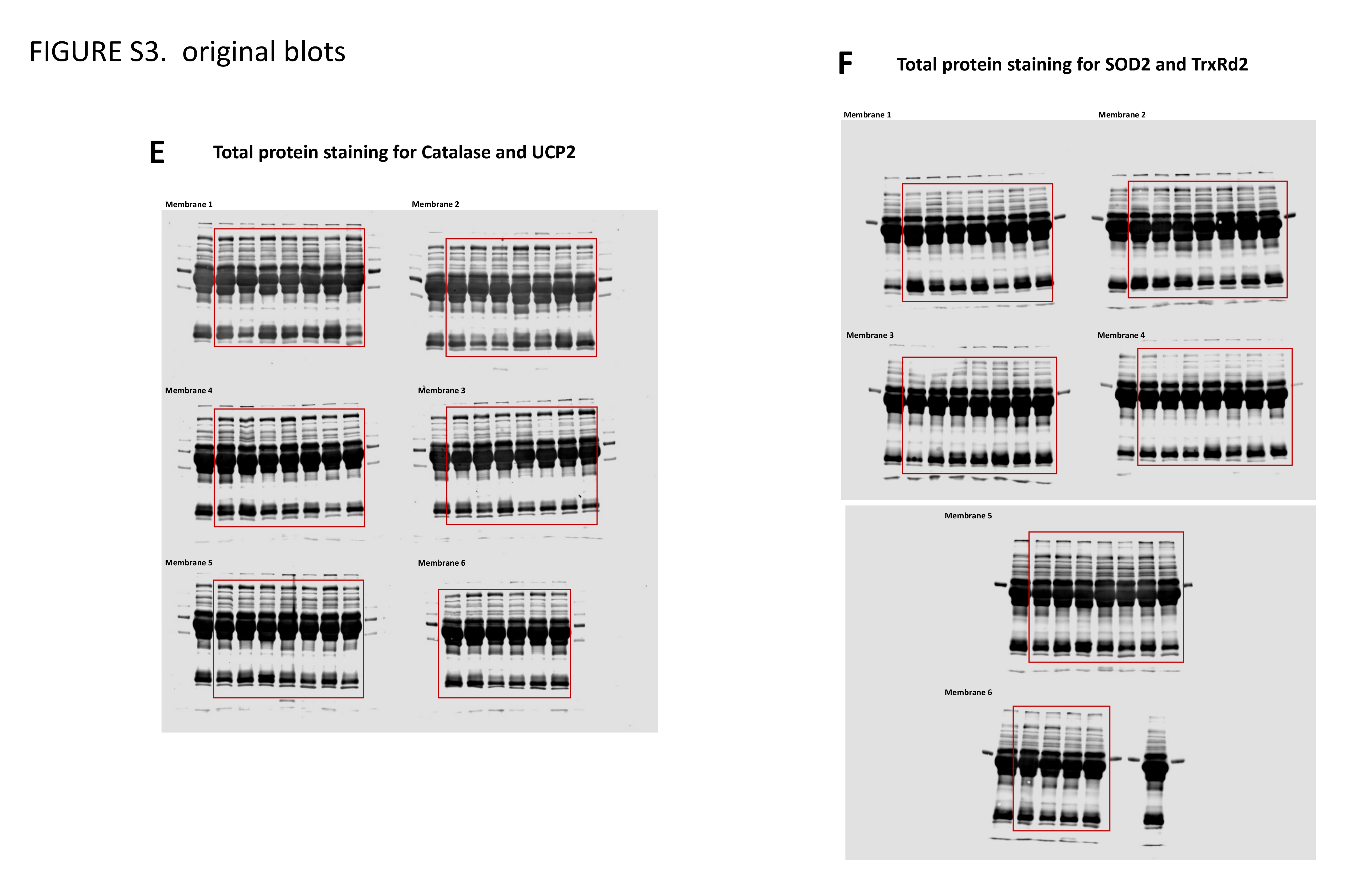
**
